# ABT-333 (Dasabuvir) Increases Action Potential Duration and Provokes Early Afterdepolarizations in Canine Left Ventricular Cells via Inhibition of I_Kr_

**DOI:** 10.3390/ph16040488

**Published:** 2023-03-25

**Authors:** Zsigmond Máté Kovács, József Óvári, Csaba Dienes, János Magyar, Tamás Bányász, Péter P. Nánási, Balázs Horváth, Adam Feher, Zoltan Varga, Norbert Szentandrássy

**Affiliations:** 1 Department of Physiology, Faculty of Medicine, University of Debrecen, H-4032 Debrecen, Hungary; 2 Doctoral School of Molecular Medicine, University of Debrecen, H-4032 Debrecen, Hungary; 3 Doctoral School of Dental Sciences, University of Debrecen, H-4032 Debrecen, Hungary; 4 Division of Sport Physiology, Department of Physiology, Faculty of Medicine, University of Debrecen, H-4032 Debrecen, Hungary; 5 Department of Dental Physiology and Pharmacology, Faculty of Dentistry, University of Debrecen, H-4032 Debrecen, Hungary; 6 Department of Biophysics and Cell Biology, Faculty of Medicine, University of Debrecen, H-4032 Debrecen, Hungary; 7 Department of Basic Medical Sciences, Faculty of Dentistry, University of Debrecen, H-4032 Debrecen, Hungary

**Keywords:** ABT-333, dasabuvir, action potential, canine, cardiomyocyte, I_Kr_, I_to_, hERG, long QT syndrome, hepatitis C virus

## Abstract

ABT-333 (dasabuvir) is an antiviral agent used in hepatitis C treatment. The molecule, similarly to some inhibitors of hERG channels, responsible for the delayed rectifier potassium current (I_Kr_), contains the methanesulfonamide group. Reduced I_Kr_ current leads to long QT syndrome and early afterdepolarizations (EADs), therefore potentially causing life-threatening arrhythmias and sudden cardiac death. Our goal was to investigate the acute effects of ABT-333 in enzymatically isolated canine left ventricular myocardial cells. Action potentials (APs) and ion currents were recorded with a sharp microelectrode technique and whole-cell patch clamp, respectively. Application of 1 μM ABT-333 prolonged the AP in a reversible manner. The maximal rates of phases 0 and 1 were irreversibly decreased. Higher ABT-333 concentrations caused larger AP prolongation, elevation of the early plateau potential, and reduction of maximal rates of phases 0, 1, and 3. EADs occurred in some cells in 3–30 μM ABT-333 concentrations. The 10 μM ABT-333-sensitive current, recorded with AP voltage clamp, contained a late outward component corresponding to I_Kr_ and an early outward one corresponding to transient outward potassium current (I_to_). ABT-333 reduced hERG-channel-mediated ion current in a concentration-dependent, partially reversible manner with a half-inhibitory concentration of 3.2 μM. As the therapeutic plasma concentration of ABT-333 can reach the low μM range, ABT-333 application carries a risk of cardiac side effects especially in case of coadministration with strong inhibitors of CYP2C8.

## 1. Introduction

The rapid component of delayed rectifier potassium current (I_Kr_) is one of the most important potassium currents of the cardiac repolarization as it corresponds to the initiation of late repolarization (phase 3) [1,2]. I_Kr_ is mediated by hERG channel proteins [3,4] and targeted by class III antiarrhythmics of the Vaughan Williams classification [5]. Early afterdepolarizations (EADs) can be generated on the basis of action potential (AP) prolongation induced by inhibition of I_Kr_ [6,7]. The AP prolongation due to reduction of I_Kr_ is known as long QT syndrome type 2 (LQTS2). This condition increases the short-term variability (SV) of APD [8] and the heterogeneity of transmural action potential duration (APD). Both of these can lead to life-threatening cardiac arrhythmias such as torsade de pointes (TdP) [9] and sudden cardiac death [10,11].

**Figure 1:**
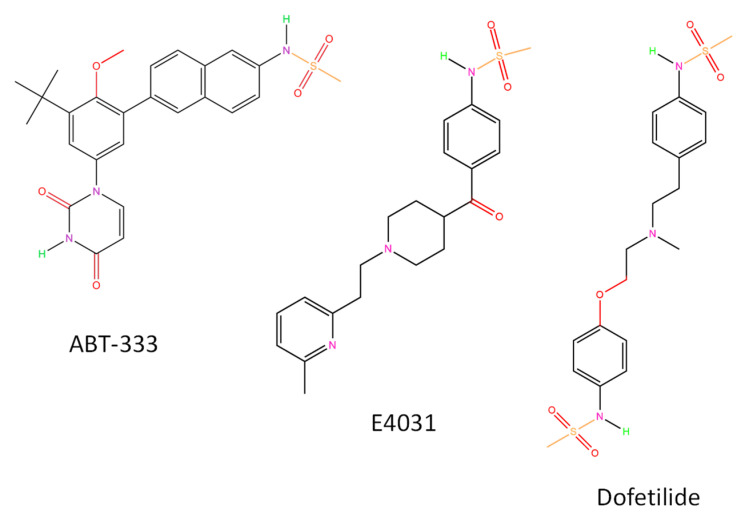
Chemical structures of ABT-333 and two I_Kr_ blockers. All structures were created by ChemDrawPro 12.0 software.

Some I_Kr_ blockers, such as E4031 and dofetilide, contain methanesulfonamide groups; there are two such groups in the latter one (Figure 1). Many active substances in the currently used medications contain the same group in their chemical structure. An example is rosuvastatin, a widely used drug to reduce serum cholesterol levels with potential I_Kr_-inhibiting action [12]. This is partly due to direct inhibition of hERG by accelerating current inactivation and also the reduction of mature hERG protein expression [13]. Two other methanesulfonamide-containing drugs are the antineoplastic amsacrine and the nonsteroidal anti-inflammatory drug nimesulide. There is no data for I_Kr_ inhibition with the latter, but amsacrine blocked hERG channels in the open and inactivated states and its binding required a common drug receptor site within the pore-S6 region [14]. Moreover, from 20 May 2018 amsacrine is listed as a drug with conditional risk of TdP (only in certain conditions) in the CredibleMeds website accessed on 11 January 2023 (https://www.crediblemeds.org/blog/five-drugs-added-crediblemeds-qtdrugs-lists).

ABT-333, or dasabuvir, a non-nucleoside hepatitis C virus (HCV) polymerase inhibitor containing the methanesulfonamide group, is used to treat chronic HCV infection, often in combination with other compounds such as ombitasvir, paritaprevir, and ritonavir [15,16,17]. Chronic HCV infection is a silent worldwide epidemic, with 130–170 million individuals involved [18] and remains quiescent for decades before significant symptoms [19]. The disease contributes to high morbidity and mortality, as well as substantial costs in healthcare [20]. Cardiac side effects of ABT-333 containing anti-HCV medication were detected in some patients, and this was summarized by Li et al. [21]. These side effects included extreme bradycardia [22], cardiac arrest [23], and chest pain [24]. As ABT-333 is metabolized by hepatic cytochrome P450 2C8 enzyme (CYP2C8), strong inhibitors of CYP2C8 may increase ABT-333 plasma levels and carry increased risk of QT prolongation [25]. This can be the case with clopidogrel administration, as its glucuronidated metabolite inhibits CYP2C8 [26].

As ABT-333 contains a methanesulfonamide group and there is a risk of QT prolongation, we aimed to study its actions on the AP of canine ventricular isolated cardiomyocytes, a good model of human cardiomyocytes regarding cellular electrophysiology [27] and also on expressed hERG channels.

## 2. Results

### 2.1. 1 µM ABT-333 Prolonged the Left Ventricular Action Potential

First, we perfused canine left ventricular cells with 1 μM ABT-333. During these experiments, ABT-333 perfusion lasted for 15 min, which was followed by a 20-minute-long washout. The analyzed parameters of the last ten action potentials recorded before ABT-333 perfusion were averaged to reduce the short-term variability of APs and are indicated as control: bicarbonate buffer containing Tyrode solution (BTY). Again, the average of the last ten APs before the washout was considered as ABT-333, while the average of other ten APs after the 20 min washout is indicated as Wo.

**Figure 2:**
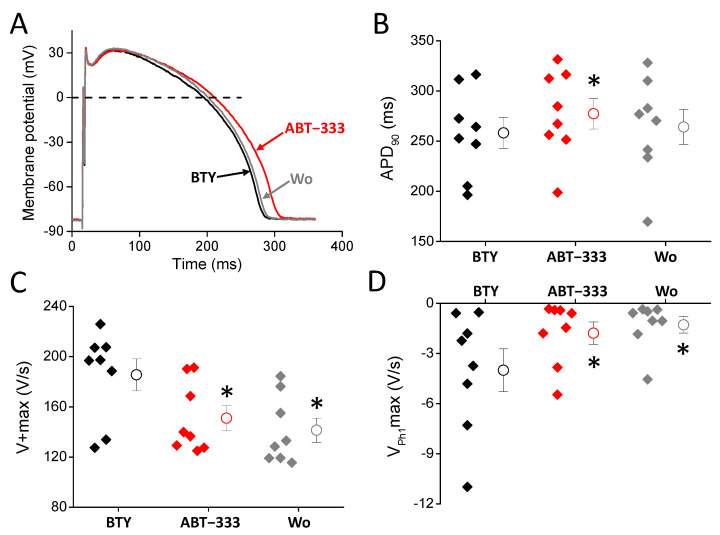
Effects of 1 μM ABT-333 on left ventricular AP. (**A**) Representative action potentials. The black trace is the AP measured in BTY (control solution), the red is the presence of 1 μM ABT-333, while the gray is after washout. (**B**–**D**) Diagrams showing AP parameters in control condition (BTY, black), in the presence of ABT-333 (1 µM, red) and after washout of ABT-333 (grey) where filled diamond symbols, circles, and whiskers show the individual values, means, and ±SEM, respectively. Values of action potential duration measured at 90% of repolarization (APD_90_, (**B**)), maximal rate of depolarization (V + max, (**C**)), and maximal rate of early repolarization (V_Ph1_max, (**D**)) obtained in 8 examined cells isolated from 4 animals. Asterisks show statistically significant difference from control (*p* < 0.05).

ABT-333, applied in 1 μM concentration, increased the AP duration of left ventricular cells from 258.3 ± 15.4 to 277.4 ± 15.3 ms (Figure 2A,B), resulting in a 7.84 ± 3.09% increase in the duration of the AP from the peak to 90% repolarization (APD_90_). This AP prolongation was not statistically significant (*p* = 0.08) for the duration of the AP from the peak to 50% repolarization (APD_50_), but the prolongation tendency was reversible upon washout, just as the ABT-333-induced APD_90_ increase. ABT-333 significantly reduced the maximal rates of phase 0 (Figure 2C) and phase 1 (V_Ph1_max, Figure 2D) to 82.8 ± 5.1 and 51.6 ± 11.3%, respectively, in a non-reversible manner. Other AP parameters, such as action potential amplitude (APA), the ratio of APD_50_ and APD_90_, overshoot potential (OSP), resting membrane potential (RMP), membrane potential differences between the RMP and the membrane potential value of at the 20 and 50% duration of APD_90_ (Plateau20 and Plateau50 amplitudes, respectively), and maximal rate of phase 3 (V − max) values did not change (Table 1).

### 2.2. Concentration Dependent Actions of ABT-333 on the Ventricular Action Potential

After detecting the 1 μM ABT-333-induced prolongation of canine left ventricular AP, we checked the actions of higher ABT-333 concentrations in a cumulative manner using 5-minute-long perfusion with increasing concentrations of ABT-333 between 1 and 30 μM. We frequently detected early afterdepolarizations (EADs) in the presence of higher ABT-333 concentrations. AP parameters of these experiments are summarized in Table 2.

**Figure 3:**
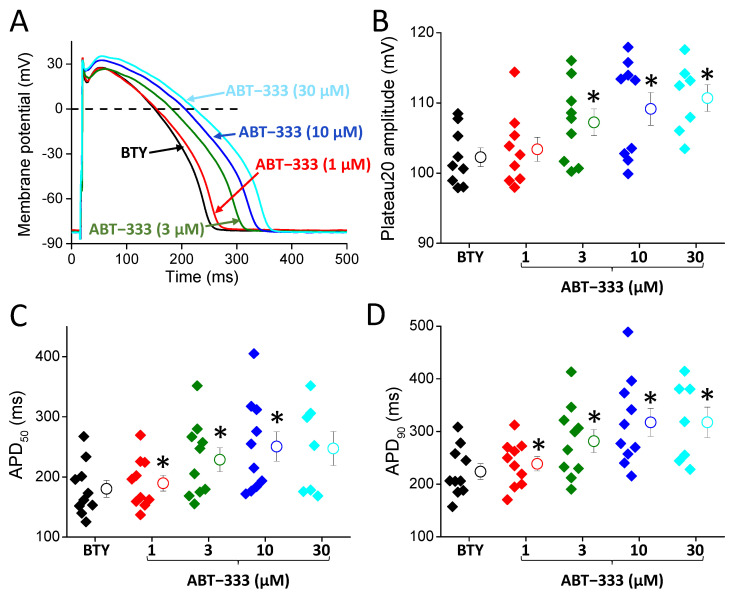
Effects of 1–30 μM ABT-333 on left ventricular AP. (**A**) Representative action potentials. The black trace is the AP measured in BTY (control solution), the color-coded ones are in the presence of increasing ABT-333 concentrations. (**B**–**D**) Diagrams showing AP parameters in the control condition (black) and in the presence of ABT-333 (various concentrations using color coding) where filled diamond symbols, circles, and whiskers show the individual values, means, and ±SEM, respectively. Values of early phase plateau amplitude (plateau20 amplitude, (**B**)), action potential duration measured at 50% of repolarization (APD_50_, (**C**)), and action potential duration measured at 90% of repolarization (APD_90_, (**D**)), obtained in 10 cells from 7 animals, except in 30 μM ABT-333, where it was 7 cells from 6 animals. Asterisks show statistically significant difference from control (*p* < 0.05).

ABT-333, applied in increasing concentrations, prolonged the AP of left ventricular cells (Figure 3A,B), resulting in 8.1 ± 2.7, 26.3 ± 5.9, 37.6 ± 7.6, and 54.3 ± 15.2% increases in APD_50_ values in cases of 1, 3, 10, and 30 μM, respectively. Similarly, ABT-333 increased APD_90_ values by 7.4 ± 2.9, 26.2 ± 6.0, 42.6 ± 8.6, and 52.6 ± 13.6% in 1, 3, 10, and 30 μM concentrations, respectively. Higher ABT-333 concentrations (3–30 μM) also increased the height of early plateau phase as plateau20 amplitude values were slightly, but significantly, increased by 4.3 ± 0.7, 6.1 ± 1.7, and 9.2 ± 1.2% in 3, 10, and 30 μM concentrations, respectively.

ABT-333 also reduced the maximal rate depolarization (phase 0; V + max), maximal rate of early repolarization (phase 1; V_Ph1_max), and maximal rate of terminal repolarization (phase 3; V − max). Only 10 and 30 μM ABT-333 decreased V + max significantly to 84.4 ± 6.2 and 77.8 ± 10.9% of the control, respectively. Higher ABT-333 concentrations also reduced V − max values to 95.1 ± 0.9, 87.1 ± 4.2, and 84.4 ± 5.6% of the control in 3, 10, and 30 μM concentrations, respectively. ABT-333 reduced the maximal rates of phase 1 repolarization to 78.5 ± 6.3, 57.7 ± 6.8, and 40.0 ± 8.3% in the cases of 3, 10, and 30 μM, respectively.

### 2.3. Development of Early Afterdepolarizations in the Presence of ABT-333

**Figure 4:**
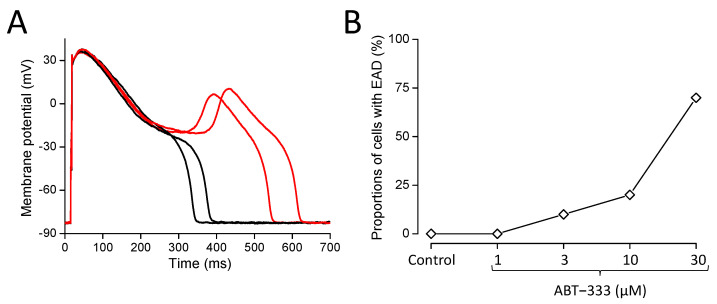
EAD formation in the presence of ABT-333. (**A**) Representative action potentials measured consecutively in the presence of 30 µM ABT-333. Red traces show APs with EADs. (**B**) Diagram shows the percentages of cells developing EADs out of the 10 studied cells obtained from 7 animals.

As mentioned previously, in the presence of ABT-333, we detected EADs in certain cells (Figure 4). We summarized the EAD appearance in a way that EAD presence was claimed in the given cell from the lowest concentration of ABT-333 in which the first EAD appeared and in all higher concentrations, even if the EADs did not persist. We did not observe any EADs in the control, and even in the presence of 1 µM ABT-333, but in higher concentrations of the drug, the percentage of cells developing EADs gradually increased (Figure 4B).

### 2.4. ABT-333-Sensitive Current Profile with AP Voltage Clamp (APVC)

**Figure 5:**
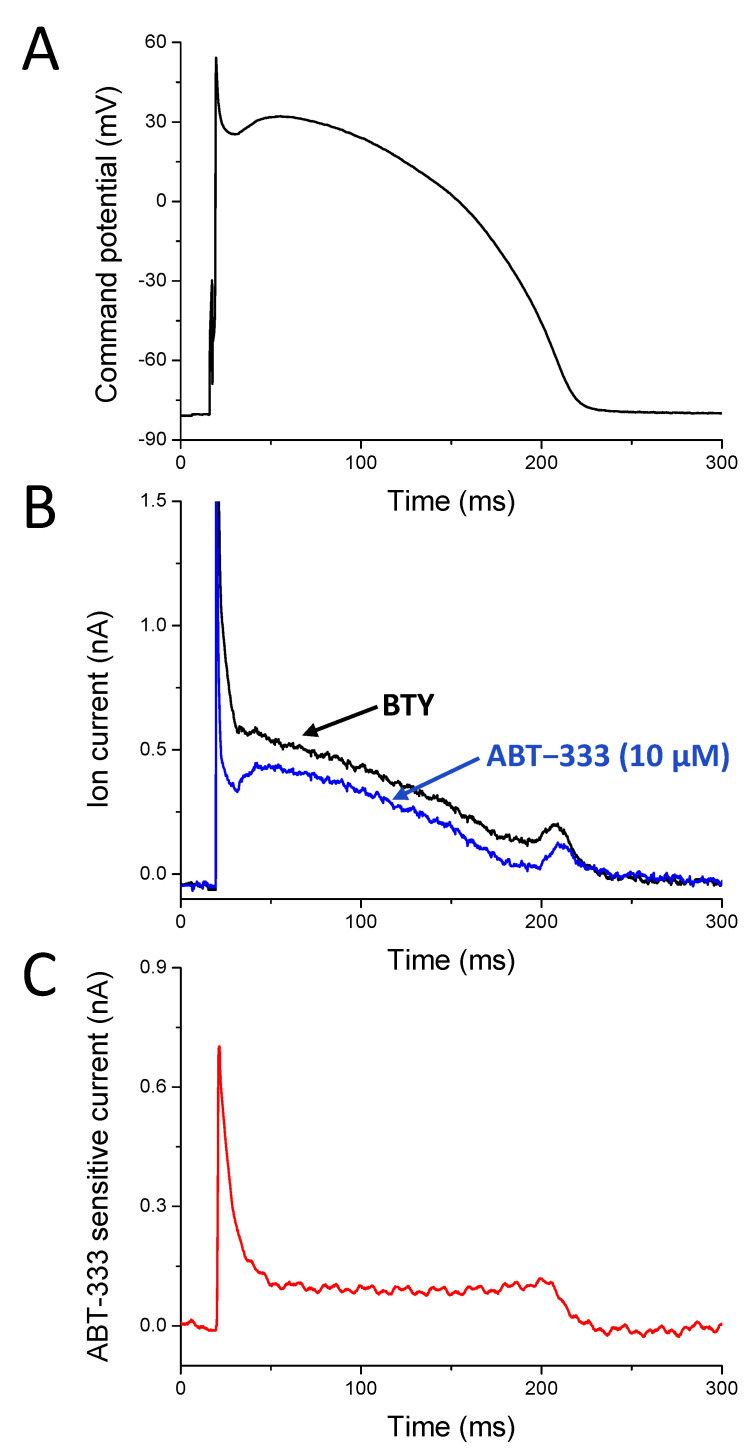
Representative APVC experiment. (**A**) The canonical action potential, which was used as stimulus command. (**B**) Current traces recorded before (black trace) and in the presence of 10 µM ABT-333 (blue trace). (**C**) The 10 µM ABT-333-sensitive current calculated by deducting the blue trace from the black one on panel B.

ABT-333-induced AP prolongation and reduction of V − max suggests the inhibition of I_Kr_ as both of these changes can be a consequence of I_Kr_ reduction. ABT-333-induced reduction of V_Ph1_max suggests inhibition of I_to_, as V_Ph1_max is greatly influenced by I_to_ amplitude. To confirm this hypothesis, we recorded the effects of 10 μM ABT-333 during the AP voltage clamp technique using a canonical AP as command potential (Figure 5A) and analyzed the properties of the ABT-333-sensitive current. The ABT-333-sensitive current was created by deducting the current trace in the presence of ABT-333 (blue trace on Figure 5B) from the one recorded before ABT-333 application (black trace on Figure 5B). Therefore, the ABT-333-sensitive current contained all those ion currents, which was modified by ABT-333 (Figure 5C). On the graph, an outward (positive) current was seen in the case of both the ABT-333-induced inhibition of an outward current as well as the ABT-333-induced increase in an inward current.

**Figure 6:**
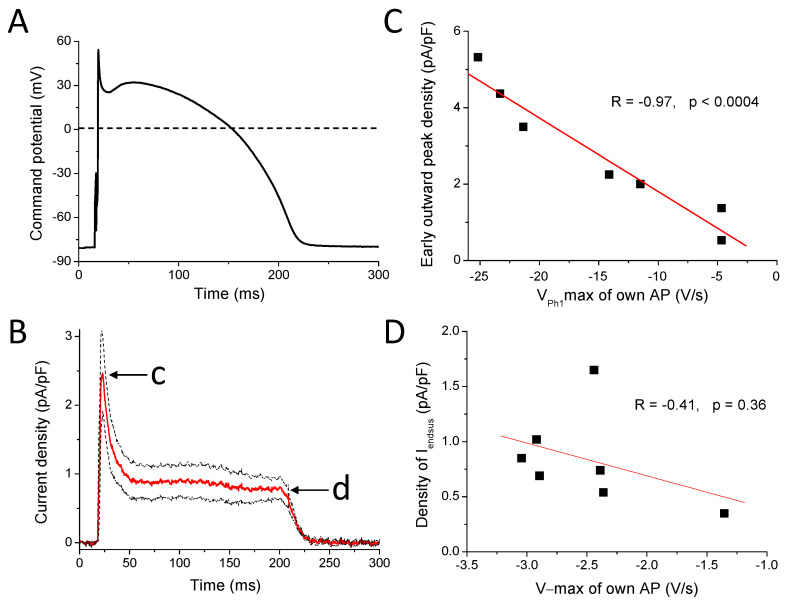
ABT-333-sensitive current in APVC condition. (**A**) The canonical action potential, which was used as stimulus command. (**B**) Average (red trace) ± SEM (black dotted lines) of 10 µM ABT-333-sensitive current. c marks the early outward peak, while d indicates the end of the sustained outward current (I_endsus_). (**C**) Correlation of early outward peak current density values (indicated with c on panel B) with V_Ph1_max of the own APs. (**D**) Relationship between density of I_endsus_ values (indicated with d on panel B) and V − max values of the own APs.

The 10 μM ABT-333-sensitive current was outward throughout the AP. We observed an early outward current peak with a density of 2.76 ± 0.64 pA/pF (Figure 6B). These current traces peaked 2.90 ± 0.81 ms later than the time of the V_Ph1_max of the command AP. On the basis of the shape of the own AP of the studied cells, there was a negative correlation between the V_Ph1_max values and the early outward peak current density values (Figure 6C). The outward current at the half duration of the command AP was 0.89 ± 0.24 pA/pF. The end of the sustained outward current (I_endsus_, measured just before the start of returning to zero, indicated with d on Figure 6B) corresponding to terminal repolarization was 0.83 ± 0.16 pA/pF, and its position was 7.82 ± 1.64 ms before the time of the V − max of the canonical AP. There was no correlation between the density of I_endsus_ and the V − max values of the own AP of the measured cells (Figure 6D).

### 2.5. ABT-333 Blocked Expressed hERG Channels in a Time- and Concentration-Dependent Manner

**Figure 7:**
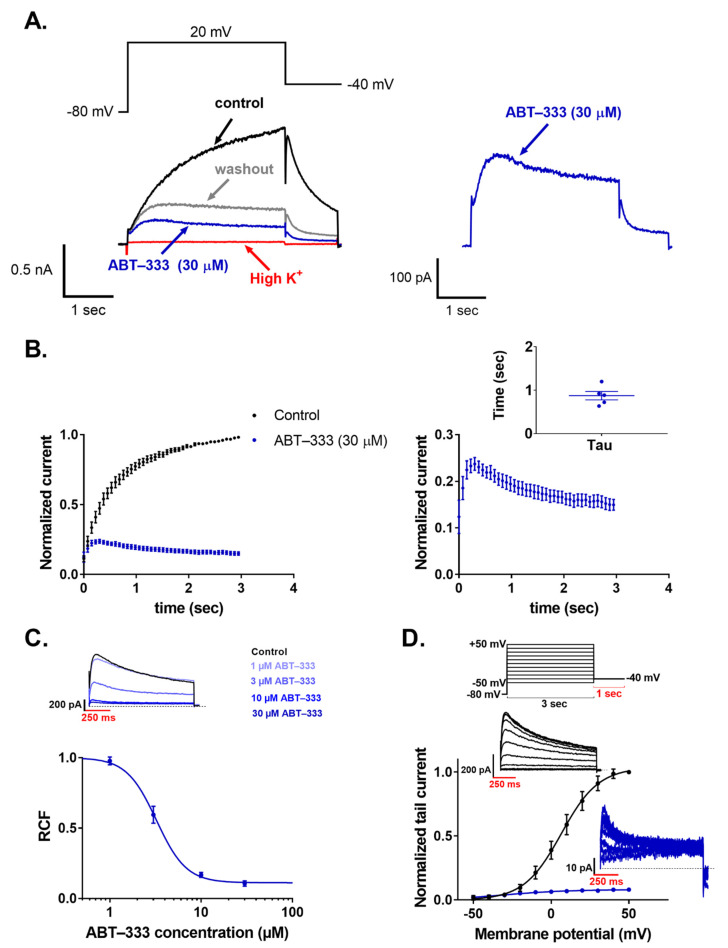
Effects of ABT-333 on hERG current expressed in HEK cells. ((**A**) **left**) A representative patch-clamp recording in voltage clamp mode. The black trace is the current measured in control solution, while the blue trace is with 30 μM ABT-333 perfusion (red trace represents perfusion-test high K^+^ application, and gray trace shows washout). ((**A**) **right**) The trace of the ABT-333 inhibited current is magnified. ((**B**) **left**) Averaged and normalized (to the peak current in control solution) current traces (*n* = 5). ((**B**) **right**) Magnified version of the ABT-333 time-dependent hERG current inhibition with individual time constants of the current decay (tau) as an inset. (**C**) Concentration–response curve of the ABT-333, which was fit on the 1, 3, 10, and 30 μM concentrations (*n* ≥ 4 for each concentration). Above the dose–response curve, a representative measurement of tail currents is shown in the control condition and during ABT-333 perfusion at the given concentrations. (**D**) G-V curves calculated from data measured in control solution (black) and in 30 μM ABT-333 (blue) (*n* = 3). Above the G-V curve, the voltage protocol is shown, and there are two representative measurements of the tail currents in control condition (black traces) and after the effect of 30 µM ABT-333 saturated (blue traces).

ABT-333 inhibited the hERG current in 30 μM concentration as observed by a 3-second-long depolarizing step to +20 mV followed by a repolarizing step to −40 mV, which led to a characteristic fast recovery of inactivated hERG channels (Figure 7A). The holding potential was −80 mV, and pulses were applied every 30 s. The application of ABT-333 caused a reduction of the hERG current both at +20 and −40 mV. The monotonic increasing control current at +20 mV changed into a decaying current in the presence of ABT-333, which was fit with a single exponential function, yielding a time constant of 0.87 ± 0.09 s (Figure 7B, *n* = 5). To test the concentration dependence of the inhibition, we applied 1, 3, 10, and 30 μM ABT-333. The remaining current fractions (RCFs) were 0.93 ± 0.04, 0.63 ± 0.05, 0.26 ± 0.04, and 0.15 ± 0.02, respectively (*n* ≥ 4 for each concentration). The concentration–response curve yielded a half maximal inhibitory concentration (IC_50_) of 3.2 μM (Figure 7C). The effect of ABT-333 on the voltage sensing of hERG channels was tested by an I-V protocol consisting of depolarizing pulses ranging from −50 mV to +50 mV in +10 mV increments. From the normalized tail current peaks, we created the G-V curves of the control solution and the 30 μM ABT-333-superfused recordings. Fitting them with the Boltzmann equation, the V_1/2_ value for the control solution was 6.50 ± 1.03 mV. For ABT-333, the V_1/2_ value was unable to be obtained this way, as its application reduced the current to the extent where proper determination of the V_1/2_ value was not possible (Figure 7D, *n* = 3). However, visually, a large voltage-shift was not apparent.

## 3. Discussion

### 3.1. Effects of 1 µM ABT-333

We first tested ABT-333 actions on cardiac AP in 1 µM, where APD_90_ was significantly and reversibly increased, likely due to the expected I_Kr_ blockade of the drug. I_Kr_ is responsible for the initiation of phase 3 repolarization, but its maximal rate (the value of V − max) mainly, and it does not exclusively depend on the density of inward rectifier potassium current, I_K1_ [1]. Accordingly, ABT-333 did not reduce V − max in 1 µM concentration, suggesting that it did not inhibit I_K1_. The APD_50_/APD_90_ ratio is often used as a marker of AP triangulation. The smaller the value, the more triangular the shape of the cardiac APs, which is often seen during inhibition of I_K1_. As the APD_50_/APD_90_ ratio did not change significantly in the presence of ABT-333, it also confirms that the drug did not inhibit I_K1_. The ABT-333-induced V_Ph1_max and V + max reductions can be caused by the inhibition of transient outward potassium current (I_to_) and sodium current (I_Na_), respectively. These actions did not reverse the 20 min washout, raising the possibility of an irreversible channel blockade by ABT-333.

### 3.2. Actions of Higher Concentrations of ABT-333

Compared to 1 µM ABT-333, higher concentrations of the drug generated further changes of AP parameters (Figure 3, Table 2). Both APD_50_ and APD_90_ values, as well as the amplitude of early plateau phase (Plateau20 amplitude), were increased in a concentration-dependent manner, suggesting again that ABT-333 reduced I_Kr_. V + max and V_Ph1_max values were also smaller in the presence of ABT-333. These observations raise the possibility of I_to_ and I_Na_ inhibition apart from I_Kr_ blockade.

At larger concentrations of ABT-333, EADs appeared, also suggesting the inhibition of I_Kr_, as AP prolongation often leads to EAD formation. In a computer simulation study, major (more than 90%) I_Kr_ reduction was needed for EAD development [6]. The APD increase, however, is not always due to I_Kr_ inhibition but can be due to the increase in sodium or calcium currents. The former one can probably be ruled out as the reduction of V + max seen with ABT-333 is not consistent with a larger sodium current. Calcium current increase by ABT-333 is a possibility, especially as it would lead to elevation of the early plateau potential, which was indeed detected with ABT-333 (Figure 3B). Therefore, it is possible that ABT-333 greatly reduced I_Kr_ or induced EADs by reduction of I_Kr_ and simultaneous increase in the calcium current, especially in higher concentrations.

### 3.3. ABT-333-Sensitive Current Profile with APVC

ABT-333-induced reduction of V_Ph1_max suggested inhibition of I_to_ on top of the I_Kr_ inhibition. We tried to confirm this with the APVC measurements. The properties of the early outward peak of the average ABT-333-sensitive current were very similar to that shown earlier with the I_to_ inhibitor 4-AP [1,28]. It was found that 1 mM 4-AP inhibits approximately 70% of I_to_ and resulted in approximately 3 pA/pF peak current density (see Figure 1 in Banyasz et al. [1]). In the current study, the ABT-333-sensitive current early peak density was slightly smaller: 2.76 ± 0.64 pA/pF, probably due to the higher presence of cells with small I_to_ current in the current study. Moreover, the time of the ABT-333-sensitive current peak was always after the time of V_Ph1_max but before the deepest point of the incisura of the command potential. Further proof that ABT-333 reduces I_to_ is the good correlation between early current peak density values and V_Ph1_max values of own APs (Figure 6C). Moreover, the early outward current decayed very rapidly, similarly to the 4-AP-sensitive current [29]. An amount of 100 µM chromanol 293B can also be used to study I_to_, wherein the decay of the current was less rapid [30]. Of note, the ABT-333-sensitive current did not decay to nearly zero, as seen with the 4-AP-sensitive one [1]. This might have been due to the effect of ABT-333 on other ion channels conducting during this early plateau phase of the AP. Nevertheless, on the basis of the results, it seems that 10 μM ABT-333 inhibits I_to_ substantially.

We also confirmed the expected I_Kr_ inhibition of ABT-333 with APVC. The position of I_endsus_ was 7.82 ± 1.64 ms before the time of the V − max of command potential, which is in good agreement with earlier results [1,29]. The peak value of the 1 μM E4031-sensitive current was approximately 0.6 pA/pF, slightly smaller than the ABT-333-sensitive current of the current study (approx. 0.8 pA/pF). This could have been due to larger I_Kr_ currents in cells of the current study than those in our previous one (see Figure 2 in Banyasz et al. [1]) as 1 μM E4031 inhibits approximately 80% of I_Kr_ [31], similarly to the large reduction of hERG by 10 μM ABT-333 (Figure 7C). In addition, it is possible that ABT-333-sensitive current might contain an activated calcium current component besides I_to_ and I_Kr_, as suggested previously. This activated calcium current, if still active during the late phase of plateau, can add to the late peak value of the ABT-333-sensitive current on top of the I_Kr_, resulting in a larger last part than the E4031 sensitive current. This possibility is evident if one observes the fingerprints of I_to_ and I_Kr_, as I_to_ decays to zero at the latest after 50 ms of the plateau phase, while I_Kr_ only starts to activate about 70–80 ms after the AP peak [1]. However, I_to_ can decay more slowly [30] and therefore might contribute to the maintained outward component of the ABT-333-sensitive current during the early plateau phase (Figure 6B). The ABT-333-induced augmentation of late sodium or calcium current can be also responsible, as that will appear as an outward current. The contribution of the late sodium current is, however, unlikely, as ABT-333 reduced the V + max value of the AP, which argues against sodium current activation.

### 3.4. ABT-333-Induced Reduction of hERG Channel Currents

ABT-333-induced AP parameter changes, namely, the increase in APD_50_ and APD_90_, mainly without the alteration of their ratio; the reduction of V − max suggested the inhibition of I_Kr_. This was expected due to the presence of the methanesulfonamide group in the structure of ABT-333. Moreover, the ABT-333-sensitive current was also outward during the late plateau phase, suggesting I_Kr_ inhibition as well. To confirm this, we carried out experiments on HEK cells stably expressing hERG channels. The hERG-mediated current was indeed reduced by ABT-333 in a concentration-dependent manner with an IC_50_ of 3.2 μM. Similarly to some AP parameters where ABT-333-induced changes were (partly) irreversible upon washout, the reduction of hERG current by ABT-333 also was unable to be fully reversed. Moreover, applying ABT-333 in 30 µM concentration caused the time-dependent inhibition during the depolarizing pulse with a time constant of 0.87 ± 0.09 s (Figure 7B). This led us to investigate the possible effects of the ABT-333 on the hERG channel gating transitions (Figure S1). On this basis, 3 µM ABT-333 significantly decreased the deactivation time constant ratio (τ_ABT-333_/τ_control_ = 0.46 ± 0.04, *p* = 0.0001), and the superfusion of 30 µM ABT-333 decreased the inactivation time constant ratio to 0.78 ± 0.15 (mean ± SEM). These results suggest that ABT-333 blocks the channel pore mainly during the open state of the channel.

### 3.5. Medical Relevance

According to our hypothesis, ABT-333 indeed blocked the I_Kr_ current. This was supported by (i) prolongation of canine ventricular AP; (ii) reduction of maximal rate of repolarization (V − max decrease); (iii) detecting I_Kr_-like profile of the late phase of the ABT-333-sensitive current with APVC; and (iv) inhibition the current of expressed hERG channels, the pore forming protein responsible for I_Kr_. In this study, we used ABT-333 between 1–30 μM. The IC_50_ value of ABT-333 against recombinant HCV polymerase (NS5B) was in the range of 2–10 nM [32]. Similar values (2–8 nM) were observed for half effective ABT-333 concentrations using cell culture assays for inhibition of replication of HCV subgenomic replicons for two genotypes (1a (H77) and 1b (Con1)), but the values were greatly (approximately 12–13-fold) increased in the presence of 40% human plasma to 20–100 nM [32]. Even more important is to compare the concentrations of ABT-333 in the current study with plasma concentrations of the drug in patients taking the compound during anti-HCV treatment. The maximal plasma concentration (Cmax) in HCV and HIV co-infected patients taking ABT-333 in combination was approximately 600 ng/mL according to King et al. [33], which corresponds to a concentration of approximately 1.2 μM. In healthy volunteers taking a 200 mg ABT-333-containing tablet alone twice daily resulted in maximal concentration (reached after 4 h of the intake) and average level after 12 h of 500 and 114 ng/mL, approximately 1 and 0.23 μM, respectively [34]. Increasing the dose resulted in linearly higher Cmax values (1.8, 2.9, and 4.2 μM for 400, 600, and 1000 mg containing tablets twice daily, respectively). There was no major daily fluctuation in plasma levels, and also in accumulation ratio, except for the 1000 mg dose where 1.6 times higher concentrations were seen by the 10th day [34]. Moreover, renal and hepatic impairment (unless severe in the case of the latter) did not alter the metabolism of ABT-333, hardly influencing the maximal plasma concentration [34]. The plasma concentration of ABT-333 was however higher in patients taking clopidogrel by approximately 2–10 times [25]. In patients taking gemfibrozil, the area under plasma concentration curve of ABT-333 was more than 10 times higher with an increase in elimination half life of ABT-333 from 5 to 90 h [35]. Taken together, it is possible that ABT-333 can reach low μM concentrations in blood plasma, given the fact that the recommended dose is 250 mg twice daily, and thereby might lead to AP prolongation. Moreover, in the case of ABT-333 overdose or coadministration with clopidogrel, gemfibrozil and other drugs impairing the metabolism of ABT-333 (exert strong blockage on CYP2C8), the arrhythmic risk of ABT-333 is even larger.

### 3.6. Summary and Conclusions

ABT-333, on the basis of its molecular structure, is expected to block hERG channels and the I_Kr_ current. This is consistent with ABT-333 application leading to a prolongation of the cardiac AP and generation of EADs. ABT-333 most likely inhibits I_to_ current too and might activate calcium current. As ABT-333 can reach low µM plasma concentrations it is possible that the abovementioned actions can develop and lead to QT prolongation or even arrhythmias especially in overdose and in patients taking ABT-333 with drugs that cause strong CYP2C8 inhibition.

## 4. Materials and Methods

### 4.1. Isolation of Canine Ventricular Myocytes

Cell isolation was carried out with the segment perfusion technique by enzymatic digestion, as described previously [2]. Intramuscular application of 10 mg/kg ketamine hydrochloride (Calypsol, Richter Gedeon, Budapest, Hungary) and 1 mg/kg xylazine hydrochloride (Sedaxylan, Eurovet Animal Health BV, Bladel, The Netherlands) was used to achieve complete narcosis in adult mongrel dogs of either sex according to protocols approved by the local ethical committee (license no. 2/2020/DEMÁB), in line with the ethical standards laid down in the Declaration of Helsinki in 1964 and its later amendments, as well as with the Guide to the Care and Use of Experimental Animals (Vol. 1, 2nd ed., 1993, and Vol. 2, 1984, Canadian Council on Animal Care). Chemicals and reagents were purchased from Sigma-Aldrich Co. (St. Louis, MO, USA) if not specified otherwise. Hearts were quickly removed in left lateral thoracotomy and washed in cold Tyrode solution containing (in mmol/L) NaCl 144, KCl 5.6, CaCl_2_ 2.5, MgCl_2_ 1.2, 4-(2-hydroxyethyl)piperazine-1-ethanesulfonic acid (HEPES) 5, glucose 10 (pH = 7.4; adjusted with NaOH). Left anterior descending coronary artery was cannulated and perfused with a nominally Ca^2+^-free JMM solution (Minimum Essential Medium Eagle, Joklik Modification, product no. M0518) gassed with a mixture of 95% O_2_ and 5% CO_2_ and supplemented with 2.5 g/L taurine, 200 mg/L NaH_2_PO_4_, 1.4 g/L NaHCO_3_, 175 mg/L pyruvic acid, 13.5 mg/L allopurinol, and 750 mg/L D-ribose, pH 6.8 at 37 °C. Then, atria were cut off, and a wedge-shaped section of the left ventricular wall supplied by the left anterior descending coronary artery was perfused. Following further 5 min of perfusion to completely remove blood from the tissue, 0.9 g/L collagenase (type II, 245 U/mg; Worthington Biochemical Co., Lakewood, NJ, USA), 2 g/L bovine serum albumin (Fraction V.), and 50 µM CaCl_2_ were added to the JMM solution. During the 30–40 min-long enzymatic digestion, the solutions were kept at 37 °C and gassed with a mixture of 95% O_2_ and 5% CO_2_. Cells were sedimented and filtered four times to remove large chunks. During this procedure, the Ca^2+^ concentration of the JMM solution was gradually restored to the final 1.8 mmol/L. After this, cells were stored in MEM solution (Minimum Essential Medium Eagle, product no. M0643) supplemented with the following: 2.5 g/L taurine, 200 mg/L NaH_2_PO_4_, 2.2 g/L NaHCO_3_, 175 mg/L pyruvic acid, 13.5 mg/L allopurinol, and 750 mg/L D-ribose (pH = 7.3, equilibrated with a mixture 95% O_2_ and 5% CO_2_) at 15 °C until further use within 36 h after isolation. The percentage of living cells (having clear cytoplasm, sharp edges, and clear striations) were usually 30–60%, and only these were used for experiments.

### 4.2. Electrophysiology

Cells were placed in a plexiglass chamber with a volume of 1 mL and continuously superfused with bicarbonate buffer containing Tyrode solution (BTY) containing (in mmol/L): NaCl 121; KCl 4; MgCl_2_ 1; CaCl_2_ 1.3; HEPES 10; glucose 10; NaHCO_3_ 25 (pH = 7.3; adjusted with NaOH) supplied by a gravity-driven system at a speed of 2 mL/min. During experiments, the bath temperature was set to 37 °C by a temperature controller (Cell Micro Controls, Norfolk, VA, USA). Cells were visualized by inverted microscopes placed in a Faraday cage on an anti-vibration table (Newport, Rochester, NY, USA). Electrical signals were recorded with intracellular amplifiers (MultiClamp 700A or 700B, Molecular Devices, San Jose, CA, USA) after analogue–digital conversion (Digidata 1440A or 1332, Molecular Devices, San Jose, CA, USA) and recorded with pClamp 10 software (Molecular Devices, San Jose, CA, USA). Cells were perfused with 1 μM ABT-333 (Cayman Chemical, Ann Arbor, MI, USA, product no. 18482) for 15 min, followed by a 20-minute-long period of washout. When higher ABT-333 concentrations were used (1-3-10–30 μM), each concentration was applied for 5 min in a cumulative manner. ABT-333 was dissolved in DMSO so that the maximal DMSO concentration (in case of 30 μM ABT-333) was 1.5%, which did not affect any of the AP parameters studied (Table S2.).

### 4.3. Recording of Action Potentials

Action potentials (APs) were measured with 3 mol/L KCl containing borosilicate microelectrodes with a tip resistance of 20–50 MΩ. A 1 s cycle length steady-state pacing was achieved for supra-threshold current pulses (2 ms long, 120–130% of threshold) produced by an electronic stimulator (DS-R3; Főnixcomp Ltd., Debrecen, Hungary). APs were digitized at 50 kHz, and upon the off-line analysis of APs, the following parameters were determined in ten consecutive APs then averaged: APD_50_ and APD_90_ values (duration of the AP from the peak to 50 and 90% of repolarization, respectively); maximal rate of phases 0, 1, and 3 (V + max, V_Ph1_max, and V − max, respectively); resting membrane potential (RMP); overshoot potential (OSP); APA (action potential amplitude, determined as the difference between OSP and RMP); and membrane potential difference between the RMP and the membrane potential value of at the 20% and 50% durations of APD_90_ (Plateau20 and Plateau50 amplitudes, respectively).

### 4.4. Action Potential Voltage Clamp (APVC) Studies

Action potential voltage clamp experiments were conducted according to the method described previously [2], and signals were digitized at 50 kHz. In the experiments, a previously recorded typical AP (recorded with 700 ms long cycle length pacing on a midmyocardial cell, termed as “canonic” AP) was applied to the voltage clamped cells as command signal. In action potential voltage clamp experiments, ABT-333-sensitive current was obtained by pharmacological subtraction calculated by deducting the current signals recorded in the presence of 10 µM ABT-333 from those measured in the control condition (BTY) [2]. On this ABT-333-sensitive current, an outward (positive) current will be seen in cases of both the ABT-333-induced inhibition of an outward current as well as the ABT-333-induced increase in an inward current.

Membrane currents were recorded using the patch-clamp technique [36] in whole-cell configuration. The cells were superfused with bicarbonate buffer containing Tyrode solution (see above for composition) at 37 °C. Borosilicate glass micropipettes had tip resistance of 2–3 MΩ after filling with pipette solution containing (in mmol/L) K-aspartate 120, KCl 30, MgATP 3, HEPES 10, Na_2_-phosphocreatine 3, EGTA 0.01, cAMP 0.002, and KOH 10 at pH = 7.3 with an osmolarity of 285 mOsm measured with a vapor pressure osmometer (Vapro 5520, Wescor Inc., Logan, UT, USA). After establishing high (1–10 GΩ)-resistance seal by gentle suction, the cell membrane beneath the tip of the electrode was disrupted by further suction and/or by applying 1.5 V electrical pulses for 1 ms. The series resistance was typically 4–6 MΩ. Experiments were discarded when the series resistance was high or substantially increased during the measurement. Ion currents were normalized to cell capacitance using short hyperpolarizing pulses to −10 mV for 45 ms from 20 ms long depolarization to 0 mV from the holding voltage of −80 mV applied at 10 Hz. The average value of cell capacitance was 154.2 ± 6.4 pF in the 7 myocytes studied.

### 4.5. Recording of hERG Currents

Whole-cell currents of voltage-clamped HEK (human embryonic kidney) cells stably expressing hERG channels were recorded by manual patch-clamp according to standard protocols using Axopatch 200B amplifiers connected to computers via Digidata 1550B digitizers (Molecular Devices, San Jose, CA, USA). Pipettes were pulled from GC 150F-15 borosilicate glass capillaries (Harvard Apparatus, Hollister, MA, USA) in three stages with 3–5 MΩ resistance. Immediately before the measurement, the cells were maintained in the recording Petri dish in bath solution (i.e., control solution, composition in mmol/L: choline chloride 140, KCl 5, MgCl_2_ 2, CaCl_2_ 2, glucose 20, HEPES 10, and CdCl_2_ 0.1; pH = 7.35 (adjusted with NaOH)). For the recordings, the composition of the solution used in patch pipette (internal solution) was made of in mmol/L: KCl 140, HEPES 10, MgCl_2_ 2, and EGTA 10, pH = 7.3 (adjusted with KOH). To test if the charge carrier was K^+^, a high concentration potassium solution (in mmol/L: KCl 150, HEPES 10, glucose 5.5, MgCl_2_ 1, CaCl_2_ 1; pH = 7.35, adjusted with KOH) was used as a positive control. ABT-333 was diluted to 30, 10, 3, and 1 μM concentrations. As ABT-333 was dissolved in DMSO, DMSO was also added to the control solution in 1.5% V/V. Solution exchange was achieved by using a gravity-flow system with continuous excess fluid removal. To avoid the changing of junction potentials during solution changes, the reference electrode, placed in a dish containing internal solution, was connected to the bath solution with an agar bridge. For the voltage-clamp measurements, a holding potential of -80 mV was used. Patch-clamp data were acquired with pClamp10 (Molecular Devices, San Jose, CA, USA). In general, currents were low pass filtered using the built-in analog four-pole Bessel filters of the amplifiers and sampled at 20 kHz. Before analysis, whole-cell current traces were digitally filtered (five-point boxcar smoothing). Experiments were performed at room temperature, ranging between 20 and 24 °C. Clampfit 10.7 (Molecular Devices, San Jose, CA, USA) and Graphpad Prism 7 (Graphpad, San Diego, CA, USA) were used for data display and analysis. The G-V curves were assembled by the normalization of the tail currents and fit with the Boltzmann equation $\left(G=1/(1+e^{\left(\left(V_{1/2}-V\right)/k\right)})\right)$ , where *k* is the slope factor, *V* is the set membrane potential, and *V*_1/2_ is the midpoint voltage. The concentration–response curve was fit with the Hill equation $\left(RCF=1/(1+({\left[c\right]}^{nH}/IC_{50}n^{H}))\right)$ , where *RCF* is the remaining current fraction, *nH* is the Hill coefficient, [*c*] is the concentration of the ABT-333, and the *IC*_50_ is the concentration where half of the channels are inhibited. The *RCF* values were calculated by the following equation: $RCF=I_{ABT}/I_{control}$ , where *I_ABT_* is the tail current measured in the presence of ABT-333, and *I_control_* is the tail current measured in control solution. The time dependence was fit with a one-phase exponential decay equation $\left(Y=\left(Y_{0}-C\right)\times e^{-t/\tau }+C\right)$ , where *Y*_0_ is the Y value when time (*t*) is zero, *C* is the plateau where *Y* saturates over time, and *τ* is the time constant.

### 4.6. Statistical Analysis

All values are presented as arithmetic means ± standard error of the mean (SEM). Given the biological variability among cells, each cell was treated as independent in the statistical tests, although more cells could be obtained from the same animal. The statistical significance of differences was evaluated using paired Student’s *t*-tests. Differences were considered significant when *p* was less than 0.05, and this is indicated with asterisks on graphs.

## Figures and Tables

**Table 1:** AP parameters obtained with 1 μM ABT-333.

Parameter	BTY (Control)	1 μM ABT-333	Washout
APA (mV)	108.9 ± 2.5	106.4 ± 3.0	105.7 ± 3.6
APD_50_ (ms)	220.0 ± 13.1	236.7 ± 13.3	225.5 ± 15.0
APD_90_ (ms)	258.3 ± 15.4	**277.4 ± 15.3**	264.2 ± 17.5
APD_50_/APD_90_	0.85 ± 0.01	0.85 ± 0.01	0.85 ± 0.01
OSP (mV)	17.6 ± 2.0	13.7 ± 3.0	12.0 ± 3.4
V_Ph1_max (V/s)	−4.00 ± 1.28	**−1.79 ± 0.67**	**−1.29 ± 0.50**
Plateau20 amplitude (mV)	106.6 ± 1.9	107.3 ± 2.4	107.0 ± 2.5
Plateau50 amplitude (mV)	90.0 ± 2.0	89.1 ± 2.3	89.2 ± 2.5
RMP (mV)	−81.2 ± 1.4	−82.6 ± 1.6	**−83.7 ± 1.3**
V + max (V/s)	185.6 ± 12.6	**151.0 ± 9.9**	**141.4 ± 9.6**
V − max (V/s)	−1.72 ± 0.08	−1.64 ± 0.07	−1.65 ± 0.08

Values in bold indicate significant difference versus BTY (control) (*p* < 0.05). Data are mean ± SEM from 8 cells obtained from 4 animals. Explanation of abbreviations can be found in the list of abbreviations.

**Table 2:** AP parameters recorded with increasing ABT-333 concentrations.

Parameter	BTY (Control)	ABT-333 (1 μM)	ABT-333 (3 μM)	ABT-333 (10 μM)	ABT-333 (30 μM)
APA (mV)	115.9 ± 2.5	115.6 ± 2.4	117.0 ± 2.6	117.5 ± 2.5	115.9 ± 1.6
APD_50_ (ms)	198.8 ± 14.4	**213.8 ± 14.2**	**252.2 ± 22.5**	**273.9 ± 25.3**	**284.3 ± 29.4**
APD_90_ (ms)	223.8 ± 14.9	**238.6 ± 13.6**	**281.7 ± 21.7**	**317.4 ± 26.5**	**317.5 ± 28.7**
APD_50_/APD_90_	0.80 ± 0.01	0.79 ± 0.02	0.81 ± 0.01	0.78 ± 0.02	0.77 ± 0.02
OSP (mV)	32.6 ± 2.7	31.3 ± 2.9	31.9 ± 2.9	33.3 ± 2.7	32.7 ± 2.5
V_Ph1_max (V/s)	−7.44 ± 1.47	**−6.69 ± 1.40**	**−5.99 ± 1.37**	**−4.21 ± 1.01**	−3.63 ± 1.15
Plateau20 amplitude (mV)	102.3 ± 1.2	103.4 ± 1.5	**106.8 ± 1.8**	**108.6 ± 2.2**	**110.7 ± 1.9**
Plateau50 amplitude (mV)	84.9 ± 1.2	84.5 ± 2.2	86.3 ± 1.5	84.4 ± 2.6	83.6 ± 2.5
RMP (mV)	−81.3 ± 1.2	−82.3 ± 1.6	**−83.0 ± 1.1**	−82.2 ± 1.2	−83.1 ± 1.8
V + max (V/s)	189.6 ± 22.6	169.5 ± 18.0	167.4 ± 15.1	**152.4 ± 14.3**	**123.5 ± 21.0**
V − max (V/s)	−1.78 ± 0.08	−1.71 ± 0.09	**−1.69 ± 0.07**	**−1.53 ± 0.07**	**−1.42 ± 0.09**

Values in bold indicate significant difference versus BTY (control) (*p* < 0.05). Data are mean ± SEM from 10 cells from 7 animals, except in 30 μM ABT-333, where it was 7 cells from 6 animals. Explanation of abbreviations can be found in the list of abbreviations.

## Data Availability

Data is contained within the article and Supplementary Material.

## Supplementary Materials

The following supporting information can be downloaded at https://www.mdpi.com/article/10.3390/ph16040488/s1, Figure S1: The effect of the ABT-333 on the gating transitions of the hERG channel; Table S1: AP parameters obtained with 1 nM ABT-333; Table S2: AP parameters obtained with 1.5% DMSO.

## Abbreviations

ABT-333	another name of the dasabuvir molecule
AP	action potential
APA	action potential amplitude
APD	action potential duration
APD_50_	action potential duration at 50% of repolarization
APD_90_	action potential duration at 90% of repolarization
APVC	action potential voltage clamp
BTY	bicarbonate buffer containing Tyrode solution
Cmax	maximal plasma concentration
CYP2C8	cytochrome P450 2C8 enzyme
EAD	early afterdepolarization
HCV	hepatitis C virus
HEK	human embryonic kidney
IC_50_	half maximal inhibitory concentration
I_endsus_	end of the ABT-333-sensitive sustained outward current
I_K1_	inward rectifier potassium current
I_Kr_	rapid component of the delayed rectifier potassium current
I_Na_	sodium current
I_to_	transient outward potassium current
LQTS2	long QT syndrome type 2
OSP	overshoot potential
V_Ph1_max	maximal rate of phase 1 repolarization
Plateau20 amplitude	difference between RMP and membrane potential value of at 20% duration of APD_90_
Plateau50 amplitude	difference between RMP and membrane potential value of at 50% duration of APD_90_
RCF	remaining current fraction
RMP	resting membrane potential
SV	short-term variability of repolarization
TdP	torsade de pointes
V + max	maximal rate of phase 0 depolarization
V − max	maximal rate of phase 3 repolarization
