# Correction: Kovács et al. ABT-333 (Dasabuvir) Increases Action Potential Duration and Provokes Early Afterdepolarizations in Canine Left Ventricular Cells via Inhibition of I_Kr_. *Pharmaceuticals* 2023, *16*, 488

**DOI:** 10.3390/ph17091137

**Published:** 2024-08-29

**Authors:** Zsigmond Máté Kovács, József Óvári, Csaba Dienes, János Magyar, Tamás Bányász, Péter P. Nánási, Balázs Horváth, Adam Feher, Zoltan Varga, Norbert Szentandrássy

**Affiliations:** 1 Department of Physiology, Faculty of Medicine, University of Debrecen, H-4032 Debrecen, Hungary; 2 Doctoral School of Molecular Medicine, University of Debrecen, H-4032 Debrecen, Hungary; 3 Doctoral School of Dental Sciences, University of Debrecen, H-4032 Debrecen, Hungary; 4 Division of Sport Physiology, Department of Physiology, Faculty of Medicine, University of Debrecen, H-4032 Debrecen, Hungary; 5 Department of Dental Physiology and Pharmacology, Faculty of Dentistry, University of Debrecen, H-4032 Debrecen, Hungary; 6 Department of Biophysics and Cell Biology, Faculty of Medicine, University of Debrecen, H-4032 Debrecen, Hungary; 7 Department of Basic Medical Sciences, Faculty of Dentistry, University of Debrecen, H-4032 Debrecen, Hungary

Text Correction

There was an error in the original publication [1]. The calculation of the therapeutic ABT-333 (dasabuvir) concentration was wrong, which changes the conclusion of the article. As the peak therapeutic plasma concentration of ABT-333 is not 1 nM, but approximately 1 µM, ABT-333 can indeed lead to cardiac side effects.

A correction has been made to the Abstract:

“… As the therapeutic plasma concentration of ABT-333 can reach the low µM range, ABT-333 application carries a risk of cardiac side effects especially in case of coadministration with strong inhibitors of CYP2C8.”

A correction has been made to Section 3.5: Medical Relevance:

“… The maximal plasma concentration (Cmax) in HCV and HIV co-infected patients taking ABT-333 in combination was approximately 600 ng/mL according to King et al. [33], which corresponds to a concentration of approximately 1.2 µM. In healthy volunteers taking a 200 mg ABT-333-containing tablet alone twice daily resulted in maximal concentration (reached after 4 h of the intake) and average level after 12 h of 500 and 114 ng/mL, approximately 1 and 0.23 µM, respectively [34]. Increasing the dose resulted in linearly higher Cmax values (1.8, 2.9, and 4.2 µM for 400, 600, and 1000 mg containing tablets twice daily, respectively). There was no major daily fluctuation in plasma levels, and also in accumulation ratio, except for the 1000 mg dose where 1.6 times higher concentrations were seen by the 10th day [34]. Moreover, renal and hepatic impairment (unless severe in the case of the latter) did not alter the metabolism of ABT-333, hardly influencing the maximal plasma concentration [34]. The plasma concentration of ABT-333 was however higher in patients taking clopidogrel by approximately 2–10 times [25]. In patients taking gemfibrozil, the area under plasma concentration curve of ABT-333 was more than 10 times higher with an increase in elimination half life of ABT-333 from 5 to 90 h [35]. Taken together, it is possible that ABT-333 can reach low µM concentrations in blood plasma, given the fact that the recommended dose is 250 mg twice daily, and thereby might lead to AP prolongation. Moreover, in the case of ABT-333 overdose or coadministration with clopidogrel, gemfibrozil and other drugs impairing the metabolism of ABT-333 (exert strong blockage on CYP2C8), the arrhythmic risk of ABT-333 is even larger.”

A correction has been made to Section 3.6: Summary and Conclusions:

“ABT-333, on the basis of its molecular structure, is expected to block hERG channels and the I_Kr_ current. This is consistent with ABT-333 application leading to a prolongation of the cardiac AP and generation of EADs. ABT-333 most likely inhibits I_to_ current too and might activate calcium current. As ABT-333 can reach low µM plasma concentrations it is possible that the abovementioned actions can develop and lead to QT prolongation or even arrhythmias especially in overdose and in patients taking ABT-333 with drugs that cause strong CYP2C8 inhibition.”

The authors state that the scientific conclusions are unaffected. This correction was approved by the Academic Editor. The original publication has also been updated.
