# Supplementary material for: ABT-333 (Dasabuvir) Increases Action Potential Duration and Provokes Early Afterdepolarizations in Canine Left Ventricular Cells via Inhibition of IKr
Source: Pharmaceuticals (Basel). 2023 Mar 25;16(4):488. doi: 10.3390/ph16040488 (PMC10143825; doi:10.3390/ph16040488)
Supplement: Supplementary file 1 [file pharmaceuticals-16-00488-s001.zip › pharmaceuticals-2208293-supplementary.pdf]

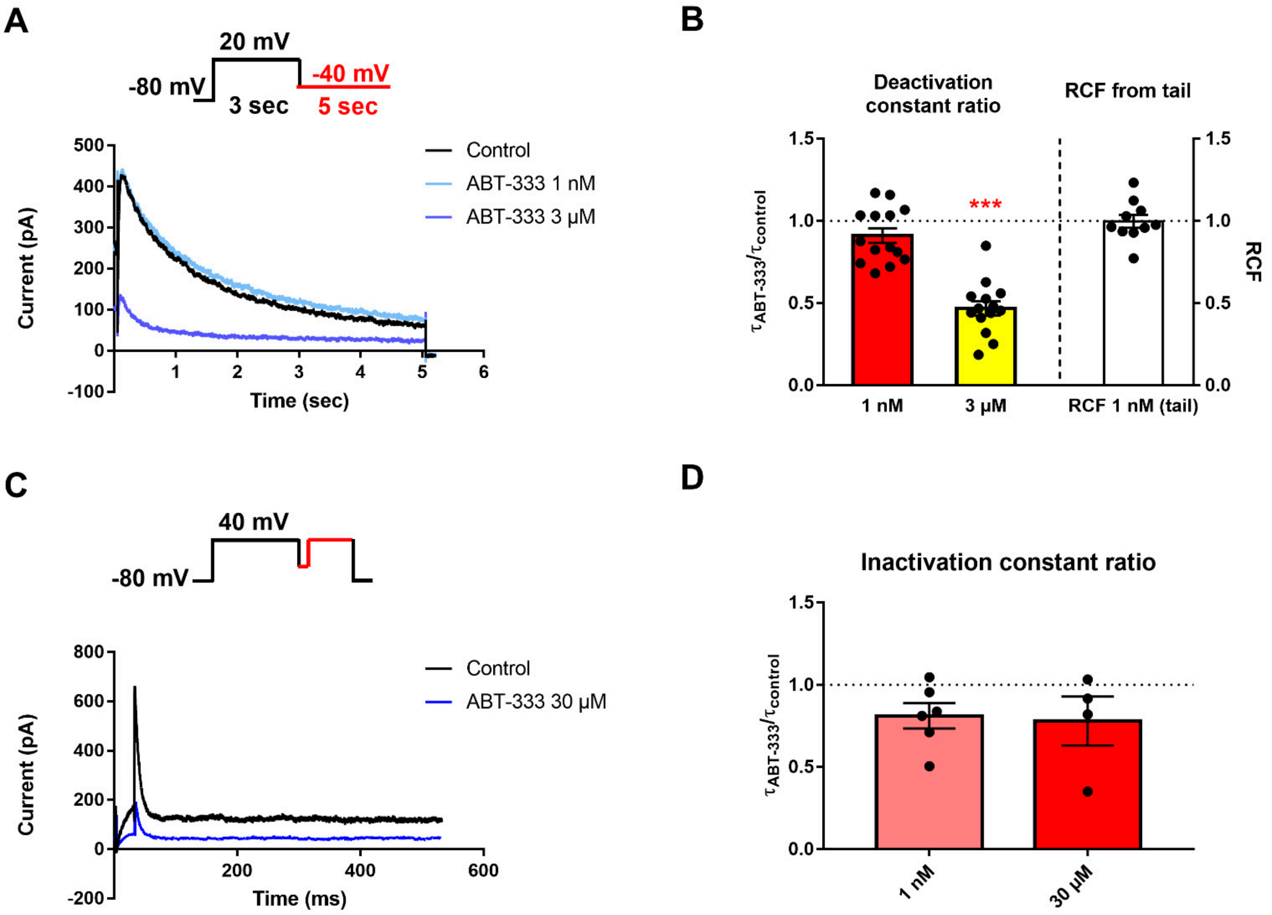

Supplementary Figure S1. The effect of the ABT-333 on the gating transitions of the hERG channel

(**A**) Representative measurement of the deactivation (O→C transition) kinetics. The applied voltage protocol is shown on the top, and the current traces below were recorded during the part of the protocol highlighted by red. The black, light blue and dark blue traces are the hERG currents control, 1 nM ABT-333 and 3 µM ABT-333 conditions respectively. (**B**) Ratios of the deactivation time constants (left) and the remaining current fraction at 1 nM ABT-333 (right). On the left y-axis the time constant ratio of the deactivation ($\tau_{ABT\text{-}333}/\tau_{control}$) is plotted (red and yellow bar charts for 1 nM and 3 µM respectively). On the right y-axis the remaining current fraction (RCF) of 1 nM ABT-333 is shown (white bar). (**C**) Representative measurement of inactivation (O→I transition) kinetics. The applied voltage protocol is shown on the top, and the current traces below were recorded during the part of the protocol highlighted by red. The black and dark blue traces are the control and 30 µM ABT-333 hERG currents respectively. (**D**) Ratios of the inactivation time constants in the presence of ABT-333. On the y-axis the time constant ratio of the inactivation ($\tau_{ABT\text{-}333}/\tau_{control}$) is plotted (pink and red bars for the 1 nM and 30 µM ABT-333 respectively). Throughout the figure, bars and error bars indicate mean ± SEM for the given number of experiments (black symbols), and asterisks show significant differences (***p<0.001).

Supplementary Table S1. AP parameters obtained with 1 nM ABT-333

Parameter	BTY (control)	1 nM ABT-333	Washout
APA (mV)	121.5±1.6	117.4±4.5	120.7±5.1
APD_{50} (ms)	168.7±12.7	157.0±11.2	142.7±27.2
APD_{90} (ms)	230.4±11.7	220.9±11.5	209.6±27.8
APD_{50}/APD_{90}	0.73±0.02	0.71±0.02	0.67±0.04
OSP (mV)	32.8±1.0	29.1±2.2	28.2±4.7
$V_{Ph1}max$ (V/s)	−10.80±3.55	−9.18±2.82	−6.95±3.28
Plateau20 amplitude (mV)	98.4±1.3	95.2±3.0	93.1±5.2
Plateau50 amplitude (mV)	80.8±1.8	77.1±3.1	74.8±5.0
RMP (mV)	−82.7±1.4	−82.3±3.2	−88.5±2.2
V+max (V/s)	305.8±15.1	253.9±22.6	258.8±14.5
V−max (V/s)	−1.25±0.12	−1.19±0.15	−1.13±0.11

Values in bold indicate significant difference versus BTY (control) ($p<0.05$). Data are mean±SEM from 5 cells obtained from 2 animals in control and ABT-333 and 3 from 1 animal for Washout. Explanation of abbreviations can be found in the list of abbreviations.

Supplementary Table S2. AP parameters obtained with 1.5% DMSO

Parameter	BTY (control)	1.5% DMSO	Washout
APA (mV)	115.9±2.0	114.3±2.4	113.1±1.6
APD_{50} (ms)	194.1±16.8	176.7±16.2	182.2±21.8
APD_{90} (ms)	250.1±16.7	238.0±15.2	240.4±21.5
APD_{50}/APD_{90}	0.77±0.03	0.74±0.03	0.74±0.03
OSP (mV)	33.1±1.8	30.9±2.0	32.4±3.8
$V_{Ph1}max$ (V/s)	−5.57±1.87	−5.43±2.01	−5.38±1.82
Plateau20 amplitude (mV)	100.2±3.2	97.2±3.5	97.9±3.6
Plateau50 amplitude (mV)	83.8±3.7	79.5±4.4	80.4±4.0
RMP (mV)	−82.8±1.8	−83.4±2.4	−80.7±3.3
SV (ms)	3.12±0.26	3.24±0.27	3.16±0.34
V+max (V/s)	201.5±18.6	175.7±28.9	**156.5±17.8**
V−max (V/s)	−1.45±0.11	−1.42±0.14	−1.36±0.11

Values in bold indicate significant difference versus BTY (control) ($p<0.05$). Data are mean±SEM from 10 cells obtained from 4 animals in control and DMSO and 9 from 4 animals for Washout. Explanation of abbreviations can be found in the list of abbreviations.
